# A systematic review of the organizational, environmental, professional and child and family factors influencing the timing of admission to hospital for children with serious infectious illness

**DOI:** 10.1371/journal.pone.0236013

**Published:** 2020-07-23

**Authors:** Bernie Carter, Damian Roland, Lucy Bray, Jane Harris, Poornima Pandey, Jo Fox, Enitan D. Carrol, Sarah Neill

**Affiliations:** 1 Faculty of Health, Social Care and Medicine, Edge Hill University, Ormskirk, United Kingdom; 2 Department of Cardiovascular Sciences, University of Leicester, Leicester, United Kingdom; 3 Faculty of Health, Public Health Institute, Liverpool John Moores University, Liverpool, United Kingdom; 4 Children’s and Adolescent Services, Kettering General Hospital NHS Foundation Trust, Kettering, United Kingdom; 5 Faculty of Health & Social Care, University of Chester, Chester, United Kingdom; 6 Department of Clinical Infection, Microbiology and Immunology, Institute of Infection and Global Health, University of Liverpool, Liverpool, United Kingdom; 7 School of Nursing and Midwifery, University of Plymouth, Plymouth, United Kingdom; RCSI & UCD Malaysia Campus (formerly Penang Medical College), MALAYSIA

## Abstract

**Background:**

Infection, particularly in the first 5 years of life, is a major cause of childhood deaths globally, many deaths from infections such as pneumonia and meningococcal disease are avoidable, if treated in time. Some factors that contribute to morbidity and mortality can be modified. These include organisational and environmental factors as well as those related to the child, family or professional.

**Objective:**

Examine what organizational and environmental factors and individual child, family and professional factors affect timing of admission to hospital for children with a serious infectious illness.

**Design:**

Systematic review.

**Data sources:**

Key search terms were identified and used to search CINAHL Plus, Medline, ASSIA, Web of Science, The Cochrane Library, Joanna Briggs Institute Database of Systematic Review.

**Study appraisal methods:**

Primary research (e.g. quantitative, qualitative and mixed methods studies) and literature reviews (e.g., systematic, scoping and narrative) were included if participants included or were restricted to children under 5 years of age with serious infectious illnesses, included parents and/or first contact health care professionals in primary care, urgent and emergency care and where the research had been conducted in OECD high income countries. The Mixed Methods Appraisal Tool was used to review the methodological quality of the studies.

**Main findings:**

Thirty-six papers were selected for full text review; 12 studies fitted the inclusion criteria. Factors influencing the timing of admission to hospital included the variability in children’s illness trajectories and pathways to hospital, parental recognition of symptoms and clinicians non-recognition of illness severity, parental help-seeking behaviour and clinician responses, access to services, use and non-use of ‘gut feeling’ by clinicians, and sub-optimal management within primary, secondary and tertiary services.

**Conclusions:**

The pathways taken by children with a serious infectious illness to hospital are complex and influenced by a variety of potentially modifiable individual, organisational, environmental and contextual factors. Supportive, accessible, respectful services that provide continuity, clear communication, advice and safety-netting are important as is improved training for clinicians and a mandate to attend to ‘gut feeling’.

**Implications:**

Relatively simple interventions such as improved communication have the potential to improve the quality of care and reduce morbidity and mortality in children with a serious infectious illness.

## Introduction

Although infection, particularly in the first 5 years of life, is a major cause of childhood deaths globally and in the UK [1], many deaths from infections such as blood stream infection and invasive meningococcal disease are avoidable, if treated in time [2]. However, differences are evident between high and low-income countries and between high-income countries; it is notable that approximately five more children die every day from avoidable causes, such as pneumonia, meningitis and septicaemia, in the UK compared to Sweden, the best in Europe [2–4].

In the UK, the latest Child Death Reviews data—all child deaths—(year ending March 2019), identified modifiable factors (factors which may have contributed to the child's death, which could potentially be modified to reduce the risk of future deaths), in nearly 4 in every 10 deaths reviewed in children aged 28 days-364 days and those aged 15–17 years [5]. The report shows that of the 75 serious case reviews (not limited to serious infectious illness) that took place, 85% were identified as having modifiable factors, a higher proportion than the 74% reported in the previous year (ending 31 March 2018) [5].

Modifiable factors which may influence timing of presentation to primary or secondary care can be related to organisational and environmental factors as well as those related to the child, family or professional. Organisational and environmental factors include: difficulties accessing primary care [6, 7]; fragmentation of services and lack of continuity in primary care [2, 8]; direct access to HCPs with paediatric training in primary care [2]; and missed opportunities for antibiotic prescribing and failure to obtain antibiotics [9]. Professional factors include the challenge experienced by some health care professionals in determining whether or not a child is seriously ill at first presentation [10, 11]. This challenge exists despite infectious illness in childhood constituting approximately 50% of children’s GP consultations and 12% of children’s hospitalisations [12]. Child and family related factors include: parents’ perception of criticism from professionals consulted [13, 14]; family past experiences of serious illness (bidirectional effect) [15, 16]; problems interpreting symptoms, assessing the severity of their child's illness and knowing when to consult [6, 7, 15, 16]. The uncertainties experienced by parents may be compounded by the repeated message for the public not to use emergency services for minor illness [17]; this may result in delayed presentation to healthcare [15] resulting in the child's illness becoming more serious than if earlier treatment had been sought.

A cohesive consideration of the literature on factors that may influence the timing of presentation is not available within the literature; this systematic review aimed to synthesise the existing evidence.

## Methods

A systematic review was undertaken to locate, appraise and synthesise evidence to answer our review question: what organizational and environmental factors and individual child, family and professional factors affect timing of admission to hospital for children with a serious infectious illness?

Note: Admission to hospital was defined as having presented to hospital and being actively investigated and treated. The conduct and reporting of this study followed the Preferred Reporting Items for Systematic Reviews and Meta-Analyses (PRISMA) guidance for systematic reviews [18]. There is no published copy of the review protocol.

### Definition of serious infectious illness

Serious infectious illness (SII) includes diagnoses such as pneumonia, bronchiolitis, meningitis, encephalitis, and sepsis. Specific criteria exist for each disease to determine its seriousness reflecting different requirements; these include identification of significant bacterial pathogen, clinical signs of sepsis, radiological confirmation plus other specific criteria [11] (see S1 File for definitions and criteria for serious infectious illness).

### Search strategy

A comprehensive search strategy with no date limits was undertaken in the following databases: CINAHL Plus with full text, Medline, ASSIA, Web of Science, The Cochrane Library and the Joanna Briggs Institute Database of Systematic Review. The search was originally undertaken in September 2015 and it was updated in March 2019 and June 2020 (last date of search 10^th^ June 2020). Both thesaurus and free text terms were searched. In order to enhance the rigour of our search, an adapted version of PICO was used to ensure that our search was directly relevant to the research question (see Table 1). Truncation and proximity operators were employed to increase the sensitivity of the search (see S2 File for Full Medline Search). Reference lists of key texts were also searched for any additional papers. Our detailed search strategy is presented in Table 2.

**Table 1:** Adapted PICO that structured the search.

PICO	Definition	Related search terms
Participants	Children with a focus on those under 5yrs old with serious infectious illness and their families	Family OR Families OR parent*OR caregiver* OR caretaker*OR carer*OR mother OR father AND Child* OR infant* or bab* or P?diatric*AND Serious infectio* OR Septi* (to capture septicaemia and septic) OR Sepsis OR Pneumonia OR Mening* (to capture meningococcal disease and meningitis) OR Encephalitis OR Respiratory
	First contact health care professionals in primary care, urgent & emergency care	General practi* (to capture general practice and general practitioners) OR Health visitor OR Paramedic*Family doctor OR Family physician OR Nurse OR Practice nurse OR Community children’s nurse OR P?diatric nurse OR children’s Nurs
Context	Primary care, first contact services, care in the home	Out of hours OR After hours Ambulatory care OR Urgent care OR Emergency care Ambulance service NHS111 OR telephone service OR telephone triage OR helpline Community OR Primary care First contact*
Interest	Factors affecting timing of admission to hospital/children’s journey	Tim* of admission OR dela* admission OR late presentation OR deter presentation OR dela* presentation Tim* of treatment OR dela* treatment OR late treatment OR earl* treatment OR timely treatment OR timely consultation OR dela* consultation Tim* of assessment OR dela* assessment OR late assessment Tim* of referral OR del* referral OR late referral Earl* diagnosis OR Late diagnosis OR Missed opportunities OR Recognition OR earl* intervention OR interpretation of symptoms OR identification of symptoms Barriers to healthcare OR access to health* Preventable OR increased OR decreased AND morbidity OR mortality Safety netting OR Information seeking OR information giv* OR Recognising symptoms OR health seeking Health service OR health systems Timely treatment OR Rapid management
Outcome	Consequences of factors affecting timing of admission such as timely treatment, early or delayed diagnosis, increased or decreased morbidity/mortality	

**Table 2:** Detailed search strategy.

Family OR families OR parent* OR caregiver* OR caretaker OR carer* OR mother OR father
**AND**
Child* OR infant* or bab* or P?diatric*
**AND**
Serious infection* OR Septi* or Sepsis OR Pneumonia OR mening* OR encephalitis OR Respiratory Severity of illness
**AND**
General practi* OR Health visitor OR Paramedic* OR Family doctor OR Family physician OR Nurse OR Practice nurse OR Community children’s nurse OR P?diatric nurse OR children’s Nurse
**AND**
Tim* of admission OR dela* admission OR late presentation OR deter presentation OR dela* presentation Tim* of treatment OR dela* treatment OR late treatment OR earl* treatment OR timely treatment OR timely consultation OR dela* consultation Tim* of assessment OR dela* assessment OR late assessment Tim* of referral OR del* referral OR late referral Earl* diagnosis OR Late diagnosis OR Missed opportunities OR Recognition OR earl* intervention OR interpretation of symptoms OR identification of symptoms Barriers to healthcare OR access to health* Preventable OR increased OR decreased AND morbidity OR mortality Safety netting OR Information seeking OR information giv*OR Recognising symptoms OR health seeking Health service OR health systems Timely treatment OR Rapid management
**AND**
Out of hours OR After hours Ambulatory care OR Urgent care OR Emergency care Ambulance service NHS111 OR telephone service OR telephone triage OR helpline Community OR Primary care First contact*
**LIMITS** Academic journals Language- English No limits set on date

### Inclusion criteria and study selection

Studies were included in the review if they fulfilled all of the criteria identified in Table 3. Our intention in focusing on high income countries was to examine a pool of articles where differences due to access to, capacity and organisation of health care were minimised. This was felt to be important as our review was Stage 1 of a larger UK-based study and we needed the findings to directly inform our research in relation to the UK health care context. A two-stage approach to screening was adopted. Stage 1 involved the independent screening of titles and abstracts (performed by JF, SN). References clearly meeting the inclusion criteria, or those where relevance was unclear, were taken forward to the next stage. Stage 2 involved the full-text screening of the studies against the inclusion criteria (performed by JF, SN, BC). We attempted to obtain full-text articles as this was seen as important in the quality appraisal process; authors were contacted to obtain the full-text articles not readily available via other sources. In order to minimize selection bias, at least two reviewers considered each paper in both stages of the screening process. If consensus could not be agreed or uncertainty existed, a third reviewer was involved in screening.

**Table 3:** Inclusion and exclusion criteria.

**Inclusion criteria** • Primary research including quantitative studies (e.g., randomized controlled clinical trials, non-randomized controlled trials, cohort studies, case-control studies, cross-sectional analytic studies, incidence or prevalence studies), qualitative studies (e.g., ethnography, phenomenology, grounded theory, and qualitative description), mixed method studies and literature reviews (e.g., systematic, meta-analysis, scoping, narrative, and integrative*)*. • Published in English • Sample including or restricted to children under 5 years of age with serious infectious illnesses • Parents/carers/ first contact health professionals • Children presenting late or who would have benefitted from earlier treatment • Research conducted in OECD high income countries. **Exclusion criteria** • Published in other languages • Exclusively about adult illnesses • Not about children with infectious illness • Children in the sample all over 5 years of age • Sample group exclusively children with HIV/ AIDS, complex or chronic childhood illness without infectious illness • Research conducted in low- or middle-income countries

### Data analysis and synthesis

A comprehensive data extraction form was developed, piloted and used to extract data specific to the aims and objectives of the review; this form aimed to provide consistency and transparency in documenting and reporting. This form was uploaded onto Google docs and two reviewers (JH and DR) extracted into the online form. This form was then exported as a.csv file and its content reviewed, refined and condensed. Table 4 (condensed summary) and S3 File: (detailed summary) present the data extraction. Due to clinical and methodological heterogeneity, a narrative approach to data synthesis and presentation was undertaken.

**Table 4:** Condensed data extraction summary.

Author, Year, Country	Study Design	No in Sample	Child age, Socioeconomic Status (SES), Disease Characteristics, Parent age/gender	Help-seeking behaviours, Organisational factors, Environmental factors, SES, Other findings.
Crocker (2013) UK [7]	**Design:** Mixed methods sequential sub sample design.	N = 151	**Child age**: 6 months-16yrs (mean 5yrs)	**Help-seeking behaviours:** Late/non-consulters significantly less likely to have taken antibiotics before presenting to hospital, & significantly more likely to have obtained advice (e.g., NHS Direct Telephone helpline) and had significantly more rapid onset of illness. Various parent factors reported (e.g., did not think earlier symptoms were serious/unusual due to child initially improving). **Organisational**: Various factors for no GP presentation (e.g. GP surgery closed). **Environmental**: Various factors for late/no GP presentation (e.g. unable to travel to GP surgery). **SES:** Late/non-consultation associated with lack of home ownership, WIMD quintile and higher ratio of children: adults in household.
			**SES:** All quintiles represented.	
			**Disease**: Community acquired pneumonia or empyema.	
			**Parent:** Carer gender not recorded	
Emery (2015), New Zealand [19]	**Design:** Mixed methods.	N = 856	**Child age**: <5yrs (mean 19mths).	**Help-seeking behaviours:** Various factors were associated with likelihood of ED presentation: increased (e.g. lower parental satisfaction scores for communication); decreased (e.g. children whose caregivers would take them back to the same doctor if still unwell). Various factors were associated with likelihood of ED admission: increased (children who had made more health professional visits before presentation); decreased (e.g., children whose caregivers would take them to a hospital ED if they had been seen the previous day by their GP and were still unwell). **Organisational:** Various factors associated with increased likelihood of presenting (e.g. GP worked ⩽20hr week) and increased likelihood of hospital admission with pneumonia (e.g., antibiotics prescribed by GP before ED presentation).
			**SES:** Measured by household deprivation score.	
			**Disease:** Pneumonia.	
Francis (2011), UK [6]	**Design:** Qualitative study.	N = 22	**Child age:** 16 months-13yrs (median 4yrs).	**Help-seeking behaviours:** All parents described potentially serious symptoms. Although most regarded these symptoms as unusual/worrying, nearly half described delay of 24h or more between first identifying the symptom(s) and consulting. Parents not consulting earlier because of a fear of ‘overreacting’, not wanting to ‘bother’ service or past experience. **Organisational (parent reported)**: Delays included difficulties with GP appointment system (e.g., prolonged waits for emergency appointments), failures/problems of appropriate triage, and failures of HCPs to respond appropriately after child had developed one or more serious symptoms.
			**SES:** Not reported.	
			**Disease**: Empyema, pneumonia, peritonsillar abscess, mastoiditis, lateral sinus thrombosis.	
			**Parent:** Mothers (n = 22), father (n = 1)	
Grant (2012), New Zealand [9]	**Design:** Case series.	N = 280	**Child age:** <5yrs (median 17mths).	**Other findings:** Receipt of antibiotic more likely if child seen by own General Practitioner (GP), less likely if the primary care clinician failed to make a diagnosis of LRTI. Mild pneumonia associated with increased likelihood of being prescribed antibiotics. Children with no opportunity to receive antibiotics had more rapidly evolving illness than those with opportunity to receive antibiotics. Various reasons for missed opportunity to receive antibiotics.
			**SES:** NZ Index of Social Deprivation.	
			**Disease**: Pneumonia.	
Kilpi (1991), Finland [20]	**Design:** Prospective cases series.	N = 286	**Child age:** 3 months-15yrs (mean 2.9yrs).	**Other findings:** Level of consciousness significantly poorer in children with short history of illness than those with long history. Seizures before or on admission were more common in the short history than the intermediate or long history groups. Children with long history of illness significantly younger than those ill for up to 48hr.
			**SES:** Not reported.	
			**Disease:** Included bacterial meningitis, haemophilus influenzae type b.	
McIntyre (2005), Australia [21]	**Design:** Case series.	N = 122	**Child age:** 1.78-179mths (median 13mths).	**Other findings:** Significant diagnostic and prognostic predictors of outcome were not having a lumbar puncture done, intensive care admission, intubation, any neurological abnormality, seizures within 48 hours, and higher temperature. The only significant therapeutic factor was administration of corticosteroids with or before antibiotics.
			**SES:** Not reported.	
Nadel (1998), UK [22]	**Design:** Prospective case note review.	N = 54	**Child age:** 1 week-15.7yrs (median 2.95yrs)	**Help-seeking behaviours:** Various reasons for some parents delaying presentation (e.g., hesitation to call GP, inappropriately reassured by advice over phone). In all cases, parents were unaware of signs of serious illness in their child. Other findings: Among children with septicaemia delay from onset until treatment initiation was longer for those who died compared with survivors.
			**SES:** Not reported.	
			**Disease:** Meningococcal septicaemia, meningococcal meningitis.	
Okike (2017), UK [23]	**Design:** Retrospective medical case note review.	N = 97	**Child age:** <90 days	**Help-seeking behaviour:** 20 parents took infants straight to the hospital, remainder phoned GP or 24-hour telephone service or contacted community midwife. Majority of parents presented to hospital within 24 hours of onset of symptoms. **Organisational:** Uncertainty in recognition, over-reliance on the presence of fever, waiting for urine samples before giving antibiotics and waiting for handover between shifts. **Other findings:** 55% infants triaged in Emergency Department during normal working hours.
			**SES:** Addressed by parental accommodation.	
			**Disease**: Included Group B strep, E Coli, other gram-negative/positive bacteria.	
			**Parent age:** (median) mothers 29yrs; fathers 32yrs.	
Thompson (2006), UK [24]	**Design:** Observational study.	N = 448	**Child age:** ≤16yrs.	**Help-seeking behaviour:** 51% of children seen by GP were sent to hospital from the 1st consultation. In most children, disease progressed very rapidly. 25% children had symptoms in the two weeks before the onset of meningococcal disease. Only 7% children had seen a doctor in the week before the onset of disease. 76.1% parents had noticed 1/more of early symptoms before hospital admission. **Other findings:** Fever was 1st symptom to be noticed in children <5yrs; headache 1st to be seen in those >5yrs. First specific clinical signs of sepsis: leg pain, abnormal skin colour, cold hands and feet, and, in older children, thirst. 1st classic symptom of meningococcal disease to emerge was rash.
			**SES:** Not reported.	
			**Disease:** Meningococcal disease	
Urbane (2019), Latvia [25)	**Design:** Prospective observational study.	N = 162	**Age**: 2mths-17.8yrs (median 43.5mths).	**Help-seeking behaviour:** 59.9% parents stated belief that fever itself is indicative of serious illness, some parents believed that other symptoms must be considered as well when evaluating the severity of illness, few parents did not believe that fever is indicative of serious illness. No association was found between the belief that fever is indicative of serious illness and parental concern. **Other findings:** The presence of clinician’s “gut feeling” was significantly more common in children who developed serious bacterial infection than in those who did not, as was “sense of reassurance” in the cases with no serious bacterial infection.
			**SES**: Not reported.	
			**Disease**: Included UTI, sepsis, pneumonia, acute osteomyelitis with bacteraemia	
			**Parent age:** Median: mothers 34yrs; fathers 33yrs.	
Van den Bruel (2012), Belgium [11]	**Design:** Observational study.	N = 3890	**Age**: 0-16yrs (mean 5.05yrs).	**Other findings:** Gut feeling that something was wrong despite clinical assessment of a non-serious illness increased risk of serious illness & acting on this feeling had potential to prevent cases being missed at cost of 44 false alarms. Compared with clinical impression that the children were seriously ill, gut feeling was consistently more specific, irrespective of the children’s age or diagnosis or the seniority of the doctor.
			**SES:** Not reported.	
			**Disease:** Pneumonia, pyelonephritis, sepsis, meningitis, cellulitis & bacterial lymphangitis.	
Young (2001), New Zealand [26]	**Design:** Qualitative.	N = 12	**Age**: <2yrs.	**Help-seeking behaviour:** Caregivers perceived themselves to be competent (e.g., prompt taking them to doctor, knew instinctively the child was unwell) but felt these subjective feelings dismissed by the doctor leading to mistrust. All parents sent home after the initial consultation but quick to return to doctor if they felt their child was not improving. Personal barriers to accessing GP existed (e.g. lack of knowledge about services, feeling dismissed as unimportant by HCPs). **Organisational**: Most caregivers visited 2 or more doctors in the community before being referred/self-referring. **Environmental:** Non-financial barriers for attending accident/medical setting rather than GP reported (e.g., GP fully booked, limited transport to GP).
			**SES**: Not reported.	
			**Disease:** Viral or bacterial pneumonia.	

### Appraisal of study quality

The Mixed Methods Appraisal Tool (MMAT): version 2018 [27] was used to review, but not score, the methodological quality of the included studies. The MMAT facilitates the critical appraisal process *"by providing*, *within a single tool*, *methodological quality criteria for different designs"* [28] p57. Five reviewers (BC, DR, SN, LB, PP) independently reviewed the extracted data and quality assessed the included studies. The quality assessment of the included studies is detailed in Table 5.

**Table 5:** MMAT synthesis.

Author	Screening questions	Criteria specific to study design					
**Qualitative criteria**							
Author (year)	Clear research questions or aims/ objectives?	Does data address research questions?	Is qualitative approach appropriate?	Are qualitative data collection methods adequate?	Are findings adequately derived from the data?	Interpretation of results substantiated by data?	Is there coherence across all stages of study?
Francis et al (2011)	Yes	Yes	Yes.	Yes	Yes	Yes	Yes
Young (2001)	Yes	Yes	Yes	Yes	CT	Yes	Yes
**Quantitative non-randomized criteria**							
Author (year)	Clear research questions or aims/ objectives?	Does data address research questions?	Are participants representative of target population?	Are measurements appropriate?	Are there complete outcome data?	Are confounders accounted for the design/ analysis?	Is intervention /exposure as intended?
Emery et al. (2015)	Yes	Yes	Yes	Yes.	Yes	Yes.	N/A.
**Quantitative descriptive criteria**							
Author (year)	Clear research questions or aims/ objectives?	Does data address research questions?	Is sampling strategy relevant?	Is sample representative of the target population?	Are measurements appropriate?	Is risk of nonresponse bias low?	Is statistical analysis appropriate?
Grant et al. (2012)	Yes	Yes	Yes	Yes	Yes	CT	Yes
Kilpi et al (1991)	Yes	Yes	Yes	CT	Yes	CT	Yes
McIntyre et al. (2005)	Yes	Yes	Yes	Yes	Yes	Yes	Yes
Okike et al. (2017)	Yes	Yes	Yes	CT	Yes.	No	Yes
Thompson et al (2006)	Yes	Yes	Yes	Yes	Yes	No	Yes
Urbane et al. (2019)	Yes	Yes	Yes	No	Yes	CT	Yes
Van den Bruel (2012)	Yes	Yes	Yes	Yes	Yes	Yes	Yes
**Mixed methods criteria**							
Author (year)	Clear research questions clear aims/ objectives?	Does data address research questions?	Is there an adequate rationale for using mixed methods?	Are different study components integrated?	Are the outputs of integration adequately interpreted?	Are inconsistencies results addressed?	Are quality criteria adhered to?
Crocker et al. (2013)	Yes	Yes	No	Yes	CT	Yes	Yes
Nadel et al. (1998)	Yes	Yes	No	Yes	Yes	No	No

Since none of the studies had a clear research question, we modified the first screening question to include clear aims/objectives. Key quality issues related to the research design not being clearly stated, challenges of recruiting representative sample, and studies being underpowered (see Table 4 and S3 File). Note: Although the two studies we have reported as mixed methods did not self-define themselves as such, they used both quantitative and qualitative (interview) methods.

## Results

**Fig 1:**
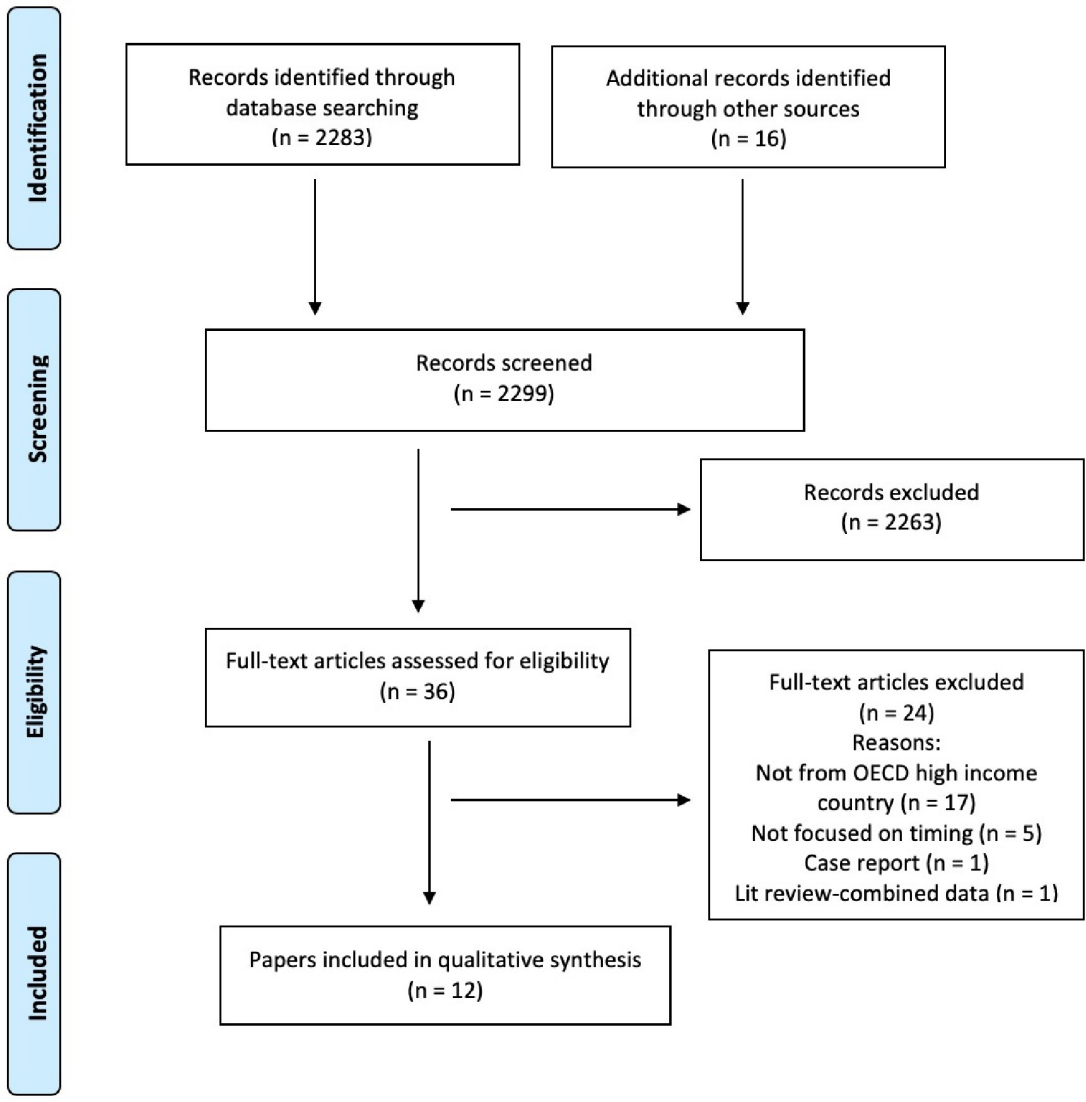
PRISMA flow diagram.

**Fig 2:**
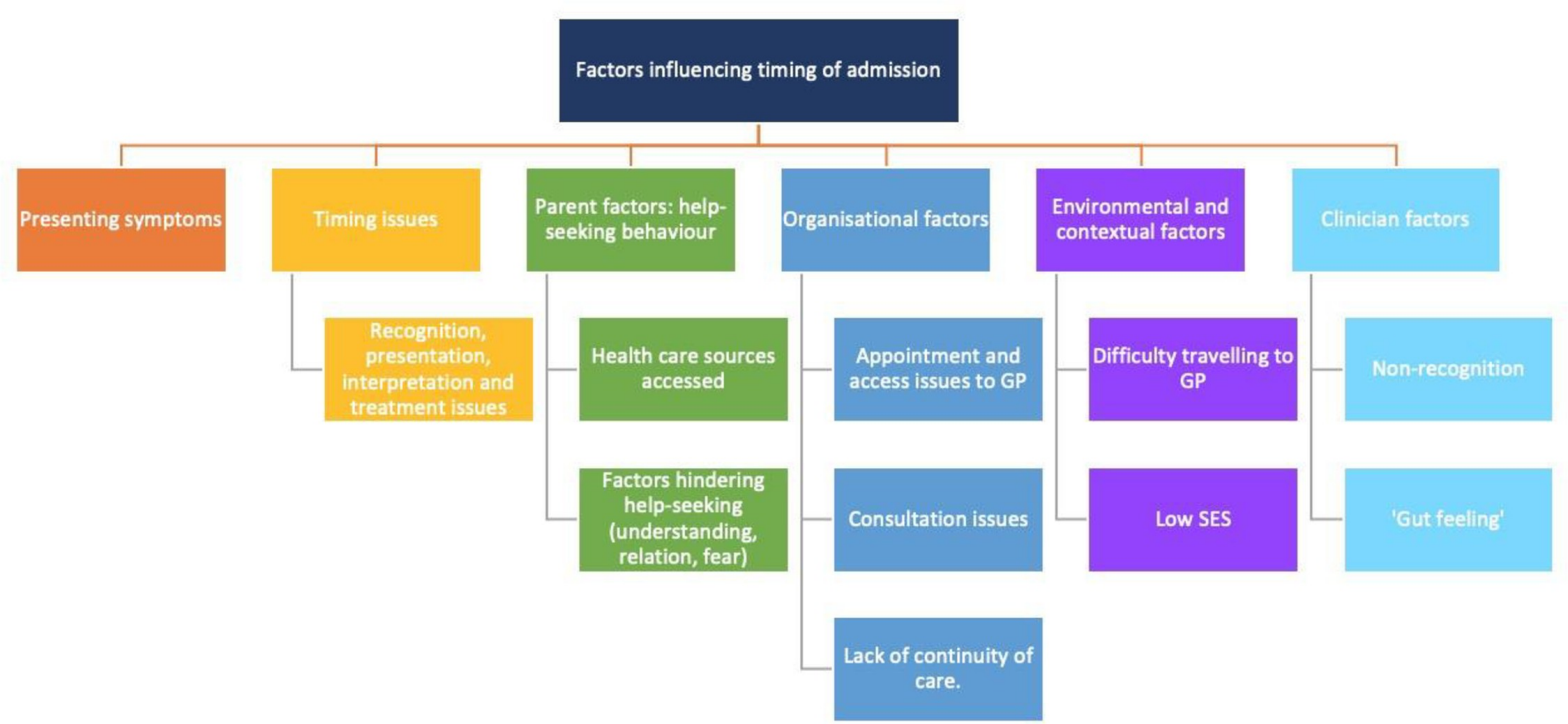
Overview of factors influencing timing of admission.

The search located 2283 references, a further 16 papers were identified from other sources and 2299 records were screened, duplicates and ineligible papers removed leaving 36 eligible for full text review which were read and assessed against the inclusion and exclusion criteria for the review; of these, 24 papers were excluded. The primary reasons for exclusion were; data not from OECD high income country (n = 17), not focused on factors influencing timing of admission (n = 5), case report (n = 1), and literature review presenting combined data (n = 1). Twelve papers were included in the review (see Fig 1 for PRISMA flow diagram). Fig 2 presents the summary of the findings.

### Overview of included studies

Data reported on studies undertaken in the UK (n = 5) [6, 7, 22–24], New Zealand (n = 3) [9, 19, 26], and one each in Finland [20], Australia [21], Latvia [25] and Belgium [11].

The studies adopted different designs; most (n = 7) used a quantitative descriptive design undertaking either prospective (n = 4) [9, 11, 20, 25] or retrospective (n = 3) [21, 23, 24] case series/case note reviews. One study used a quantitative non-randomised (case control) design [19], two used a mixed methods design (reviewing the case notes and undertaking interviews) [7, 22] and two used a generic qualitative design [6, 26]. Typically, the case series/case note reviews used a questionnaire as well as reviewing case notes. None of the studies reported the theoretical perspective underpinning their work. Two studies did not report ethics review approval [20, 26].

All study populations comprised children and/or young people aged 1 week to <18 years; typically, under 5 years of age. Some studies recruited parents [7, 9, 20, 22, 23, 26] or clinicians [11, 21] or both [19, 24, 25].

The presenting disease fell into three categories: pneumonia (n = 6) [7, 9, 11, 19, 26]; meningococcal disease (n = 5) [20–24]; other disease (n = 2) [6, 25].

Recruitment was undertaken from a secondary care/hospital setting (n = 9) [6, 7, 9, 19, 20, 22, 25, 26]; primary care setting (n = 1) [11], both hospital and primary care (n = 1) [24], via a register/database (n = 2) [21, 23]. The number of participants included in the studies ranged from 12 [26) to 3890 [11].

Socioeconomic status (SES) was directly measured using validated tools in three studies [7, 9, 19], indirectly in two studies [6, 23] and not reported in the remaining studies (n = 7).

Our assessment of study quality based on our use of MMAT (Table 5) showed that most of the studies recognised limitations, mostly related to the study population. These included: invitation and/or selection bias (n = 3) [7, 22, 25]; excluded populations (n = 4) [6, 7, 9, 25]; uneven distribution [19]; seasonal bias [19]; response bias [7]; recall bias (n = 5) [9, 11, 22–24]; reliance on retrospective data [6, 24]; incomplete data [21]; and study being underpowered [11].

### Presenting symptoms

A range of presenting symptoms (respiratory, neurological, systemic) were reported within the studies. Some symptoms were specific for meningococcal disease or pneumonia. A limited number of symptoms were reported for children presenting with either meningococcal disease or pneumonia; these were increased respiratory rate, breathing difficulties; vomiting, poor feeding; and increased sleeping, lethargy, drowsiness [9, 24]. The other presenting symptoms reported for pneumonia were cough, wheeze, noisy breathing, irritability [9]. The other presenting symptoms for meningococcal disease were leg pain [24], level of consciousness [20], fever [23, 24], seizures [20, 23], abnormal skin colour, thirst, and rash [24]. Most symptoms occurred before first medical contact [24].

### Timing-related factors

Delays occurred within both home and primary care settings. Delays in presentation to either primary or secondary care were associated with issues related to recognition, presentation, interpretation and treatment (primarily administration of parenteral antibiotics) [7, 22, 23]. Delays were associated with worse outcomes [21–23]. For children with septicaemia the delay from onset was longer for those who died compared with survivors [22]. Delay between presentation and initiation of treatment for meningitis was 2–12 hours (9 children had repeat visits to GP, 7 taken to A&E without further attempts to see GP) [22]. Median time from onset of first feature to first help in infants (meningitis) was 5 hours and this median was longer for infants with poor outcomes than those who recovered without sequelae [23]. There was close correspondence between median time of onset of meningococcal rash and first medical contact [24]. Findings from one study showed that children with a long history of meningitis (>48hr) did significantly better than those with a shorter history as judged by clinical and laboratory variables [20].

### Parent factors: Help-seeking behaviour

Help-seeking behaviours in which parents engaged with health services were described in eight studies [6, 7, 19, 22–26]; parents of younger children were more likely to consult than those with older children [7]. Parents sought help for their sick child from a range of different health care sources including A&E, urgent care, GP, 24hr NHS direct line telephone service, midwife, website [7, 23].

Broadly those factors that hindered initial and ongoing help-seeking could be placed into three categories: understanding, relational, and fear related. Issues related to understanding included parents/carers not recognising their child’s symptoms as being problematic and therefore not seeking help from a GP [7], difficulty in assessing and/or interpreting their child's condition [6, 22], and poor understanding of their child's illness post-consultation [6], belief that fever is key indicator of serious illness [25]. However, some mothers reported being confident in their ability to instinctively distinguish serious from non-serious illness, drawing on 'mothers instinct' [26]. The relational issues reflected a mistrust of doctors [26], a perceived inability to challenge clinicians' ideas [6] and past experience indicating they would not be believed [6, 26]. Fear-related issues included fear of bothering GP or wasting GP's time [6, 7], fear of over-reacting or appearing neurotic [6], hesitancy to call GP at weekend/night [22]. Some parents were concerned about antibiotics being prescribed [6].

### Organisational factors

Organisational factors which did not facilitate review by the GP included surgery being closed [7], delays or difficulties getting an appointment with GP [6, 7], and GP declining to do a home visit [7]. Lack of knowledge of services was identified [26]. Consultation issues included rushed consultations [6], the use of over the phone communication for diagnosis and treatment [6], contradictory information from different clinicians [26], and failure in triage in GP setting [6]. Lack of continuity of care such as the child not having a single identified GP or their GP working ⩽ 20 hours a week [19] were also identified as factors.

### Environmental and contextual factors

Environmental factors which did not facilitate ‘timely’ review by the general practitioner included difficulty travelling to GP for appointment (e.g., no car or child too ill). Some families used A&E in preference to a GP [7], for some because it was closer than the GP surgery [26]. Low SES, as indicated by WIMD quintile, lack of home ownership, and higher ratio of children to adults in household, was associated with parents who were late or non-consulters with their GP before their child was hospitalised [7]. Households in SES deprived areas were noted to be over-represented [9].

### Clinician factors: Non-recognition

Non-recognition of (and therefore treat or refer) serious illness was a factor for clinicians in primary care, even in the presence of fever, petechiae/purpuric rash and other clinical features of serious illness [22]. Some GPs were not aware that the presence of diarrhoea does not rule out sepsis [11].

### Clinician factors: 'Gut feeling'

Two studies addressed clinician 'gut feeling' that something was ‘wrong’ and its added value in identifying serious illness [11, 25]. In one study 'gut feeling' was not significantly predictive of the child being diagnosed with serious bacterial infection (SBI), although it was more commonly related to children who did, rather than did not, develop SBI [25]. The other study showed that despite the clinical assessment of non-severe illness, 'gut feeling' was found to be linked to the risk of serious illness; they also found that acting on this ‘gut feeling’ had the potential to prevent two of the six cases being missed at the cost of 44 false alarms [11]. ‘Gut feeling’ was most likely to be triggered by history of convulsions, parental concern and the child's appearance, pattern of breathing and level of drowsiness was also significant [11]. In children whose pattern of breathing and level of consciousness were indicative of clinical concern, clinician's 'gut feeling' was more likely to be provoked by parental concern [11]. GPs did not always act on their gut feeling that the child was seriously ill; 4/21 children admitted to hospital were not referred at first presentation despite the presence of ‘gut feeling’ [11]. These children did not differ significantly from those who were referred. ‘Gut feeling’ was relied on less as the clinicians gained experience [11].

## Discussion

This review supports existing evidence that the pathway to hospital is complex and modifiable factors exist that are amenable to intervention [29].

### Nature of illnesses and trajectories

Typically, the studies addressed pneumonia and meningococcal disease and the presenting children were young (<5yrs), even in those studies including older children. The trajectories were variable even within the same diagnostic category, some trajectories were influenced by the child’s age with, for example, the median time between onset and admission to hospital for meningococcal disease being 13 hours in children younger than 1 year when compared to 22 hours in children aged 15–16 years [24]. Some children had been symptomatic two weeks before the onset of meningococcal disease, although few had seen their GP for these initial symptoms [24]. This variability [24], as seen with other diagnoses, is an important element for clinicians to be aware of when a child presents to them.

The children’s journeys to hospital took different routes with some parents going directly to A&E or urgent care services [22, 23] whilst others sought help from their GP or midwife or via 24hr NHS direct telephone service or website [7, 23]. Some children were taken to their GP on more than one occasion [22, 26] indicating a persistence of parental concern which was not always acknowledged by the health professional. Considering evidence that a significant proportion of attendances to the emergency department are appropriate [30], a greater appreciation of parental concern by health professionals could enhance timely referral. Parental concern is included in the assessment algorithms of NICE guidelines on sepsis and meningitis [31, 32].

### Factors affecting timing of admission to hospital

A range of different factors influence the timing between presenting symptoms, parent raising concern that their child was sick and the point at which the child was admitted to hospital and was receiving active assessment and intervention.

#### Parents recognition of symptoms

Parental recognition of symptoms was identified as being problematic with some parents reporting difficulty in recognising and assessing their child's symptoms [6, 7, 22], and noting that this difficulty could persist post-consultation [6]. Other studies have identified similar difficulties for parents in relation to interpreting symptoms and severity and when it is appropriate to seek professional support [15]. There is clearly the potential for improved parental education [6] and carefully worded advice as this would act as a means of informing parents’ understanding of key symptoms and act as 'safety net' [11, 33] or promote parents’ intent to reconsult [34]. Safety netting, within the healthcare context refers to the ‘provision of information to help patients or carers identify the need to consult a healthcare professional if a health concern arises or changes’ [35]. However, developing robust information resources that meet the different health literacy requirements and preferences of parents is not without challenge [36]; simply supplying written information neither guarantees understanding nor engagement [35]. Even those parents who were confident in their instinctive sense of the seriousness of their child’s condition were not always able to convince the GP that their child was sick [26] as seen in other published work [37].

#### Help seeking behaviour

There is a wealth of literature on the inappropriate use of services [38–40] and often with a focus on parents’ health literacy [41]. However, there is a small but growing body of literature on parents’ reluctance to ‘bother’ the doctor or waste service time [14, 15] or hesitation to make contact at the weekend or night time. Although there was some evidence of this in the review [6, 7, 22], the legitimacy of demand is dependent on context [42]. Some parents delayed any face-to-face contact as they had been reassured via telephone contact [22]. Other parents chose to access services such as urgent care or out of hours services [23] in preference to accessing their child’s GP (e.g., out of hours services). This has been reported in other studies where reasons similarly included perceived ease of access and/or concern about the severity of the child’s symptoms [34, 43]. Other parents were reluctant to engage with the GP due to previous poor experience such as being criticised or dismissed or feeling uncomfortable [6, 26] as also seen in other studies [14, 44, 45]. Other parents were unsure about the acceptability of returning to primary care [26] if they remained concerned about their child’s condition. Recognising parental expertise [37, 46], empowering parents to contradict clinicians [22], establishing and sustaining trust [26] and creating supportive conditions for parents to be able to seek help from their GP or other services early in their child’s illness course and to know when to reconsult if they child’s illness progresses [33] has the potential to positively influence the child’s journey to hospital. Better understanding of doctor-patient relationship, particularly for different SES/ethnic groups [19] is an important component to consider in enhancing service provision.

#### Access to services

Delayed diagnosis predicts morbidity [21] as does rapid disease trajectory [20]; the latter being a reminder that not every child’s poor outcome or child death can be prevented. However, promoting easier access to less fragmented services, avoiding problems such as not being able to get a GP appointment or being able to access out-of-hours consultation and lack of continuity in primary care need to be addressed [2, 8, 43]. Some general practice emergency access systems may not work as well as they should in achieving sensitivity identifying those developing complicated respiratory tract infections [6]. Caregivers should be instructed on what to do outside the opening hours of the family practice if the child's condition worsens [26], this information should form part of the safety net of information given to parents [35].

#### Clinician related factors

The challenge of diagnosing children whose condition is likely to deteriorate is complex and clinicians in primary and secondary care need to have clearer information, guidance (e.g., an algorithm, or need to follow algorithms/protocols provided) [31, 32, 47, 48] and messages about signs of serious illness (red flags) to avoid missed recognition and the importance of not delaying treatment [6, 22, 49]. Findings suggest that there is potential benefit in considering shifting the focus from classic symptoms to early recognition of sepsis [24].

Evidence from the studies focusing on clinician ‘gut feeling’ of something being wrong reveals contradictory findings. In our review one study demonstrated a link between ‘gut feeling’ and a child’s risk of serious illness [25] whilst the other noted ‘gut feeling’ was not significantly predictive of SII [11]. However, it is important to recognise that contradictions may arise from factors such as differences in the settings, experience of the clinician, differences in prevalence of serious infection and availability of continuity of care. Such factors may have influenced the statistical power of the prognostic value of “gut feeling”. Other studies considering ‘gut feeling’ or ‘clinical impression’ in relation to the assessment of the acutely ill child, emphasise its value in clinical prediction that ‘something is wrong’ [50, 51]. However, despite a contradictory evidence base, it is hard to ignore the proposition that a clinician's ‘gut feeling’ should make three things mandatory: carry on careful examination, seek more experienced advice, and give parents carefully worded advice to act as safety net [11]. These three mandates are not onerous and have the potential to both reassure parents and save lives.

Considering the challenge/failure of diagnosis and suboptimal management of some children, it appears that improvements could be made in various ways, including enhancing the skills of primary and secondary care clinicians through improved and ongoing training [6, 47] such as that provided by www.spottingthesickchild.com, ensuring junior paediatricians receive advanced life support training [22], improved supervision from consultants [47] and more robust documentation of the child's symptoms and condition at each stage of the journey, in primary [9] and secondary [47] care. Improving relational continuity also has the potential to improve the recognition of deterioration as the clinician will able to augment the written record with memories of preceding encounters with the child.

### Strengths and limitations

This systematic review used a robust and iterative methodological approach and included an analysis of study quality. The number of studies included was small and the methodological approach and focus of the studies and diagnosis of child was diverse, thus making it challenging to draw clear conclusions. Although poor reporting quality (e.g. research questions not reported) was evident in some of the studies, overall, the quality of the studies was sound with most studies clearly identifying limitations relating to their study population. A common limitation was that many studies had reporting or recall bias. Recruitment is likely to have utilised both families and parents with significant concerns or those who came to harm, so are not representative. Some studies were underpowered. Predominantly data collection was retrospective, and while this was often a necessity due to study design, it was difficult to evaluate how this may have impacted on interpretation in many studies.

The term ‘admission to hospital’ is rarely clarified meaning comparison across studies is difficult. Our decision to include a wide age range rather than concentrating solely on children under the age of 5 reduces the focus on the age group most typically presenting. Some findings such as the likelihood of admission with pneumonia being increased when antibiotics were prescribed by GP before admission [22], perhaps are less applicable since the introduction of pneumococcal vaccine.

### Implications for practice

In summary, supportive, accessible, respectful services that provide continuity, clear communication and advice are important and have the potential to reduce the reasons why some parents may hesitate to seek or continue to seek help. High quality training and support for clinicians to spot the sick child and encouragement to attend to ‘gut feeling’ have the potential to improve identification of the sick child within any of the settings where a child presents. Improved parent-facing information that recognises the diversity of health literacy should be available to inform parents and clearly instruct them how to act.

### Directions for further research

Our findings indicate that further research is needed to better understand the doctor-patient relationship, in particular to identify the sources of perceived criticism and how such criticism can be reframed as helpful advice. Further research on parental concern and how to recognise it would enhance health professionals’ ability to recognise important symptoms and enhance timely referral.

## Conclusion

Our conclusions need to be considered in relation to the limitations of the studies reviewed and the risk of bias we have previously noted. We found reasonably robust evidence that both clinician-related and parent-related factors impact on the timeliness of a child’s journey to hospital but less depth of cohesive evidence in relation to environmental and organisational and contextual factors. However, where the evidence exists these factors seem inextricably linked.

## Supporting information

S1 File Definitions of serious infectious illness, developed from [11, 52, 53]. (DOCX)

S2 File Search conducted in MEDLINE via EBSCOHost. (DOCX)

S3 File Detailed data extraction summary. (DOCX)

## References

[1] Roser M, Ritchie H, Dadonaite B. Child & Infant Mortality.; 2020.

[2] Wolfe I, Cass H, Thompson MJ, Craft A, Peile E, Wiegersma PA, et al Improving child health services in the UK: insights from Europe and their implications for the NHS reforms. BMJ. 2011;342(mar08 1):d1277–d. 10.1136/bmj.d1277 21385800

[3] Wolfe I, Donkin A, Marmot M, Macfarlane A, Cass H, Viner R. UK child survival in a European context: recommendations for a national Countdown Collaboration. Archives of Disease in Childhood. 2015;100(10):907–14. 10.1136/archdischild-2014-306752 25957319

[4] Wolfe I, Thompson M, Gill P, Tamburlini G, Blair M, van den Bruel A, et al Health services for children in western Europe. The Lancet. 2013;381(9873):1224–34. 10.1016/S0140-6736(12)62085-6 23541056

[5] Community and Mental Health Team ND. Child Death Reviews: NHS Digital; 2019 [Available from: https://digital.nhs.uk/data-and-information/publications/statistical/child-death-reviews/2019.

[6] Francis NA, Crocker JC, Gamper A, Brookes-Howell L, Powell C, Butler CC. Missed opportunities for earlier treatment? A qualitative interview study with parents of children admitted to hospital with serious respiratory tract infections. Archives of Disease in Childhood. 2011;96(2):154–9. 10.1136/adc.2010.188680 21047831

[7] Crocker JC, Evans MR, Powell CVE, Hood K, Butler CC. Why some children hospitalized for pneumonia do not consult with a general practitioner before the day of hospitalization. European Journal of General Practice. 2013;19(4):213–20. 10.3109/13814788.2013.795538 23815375

[8] Parliamentary and Health Service Ombudsman An avoidable death of a three-year-old child from sepsis. A report by the Health Service Ombudsman for England on an investigation into a complaint from Mr and Mrs Morrish about The Cricketfield Surgery, NHS Direct, Devon Doctors Ltd, South Devon Healthcare NHS Foundation Trust and NHS Devon Plymouth and Torbay Cluster. London; 2014.

[9] Grant C, Harnden A, Mant D, Emery D, Coster G. Why do children hospitalised with pneumonia not receive antibiotics in primary care? Archives of Disease in Childhood. 2012;97(1):21–7. 10.1136/archdischild-2011-300604 22100740

[10] Buntinx F, Mant D, Van den Bruel A, Donner-Banzhof N, Dinant G-J. Dealing with low-incidence serious diseases in general practice. British Journal of General Practice. 2011;61(582):43–6. 10.3399/bjgp11X548974 21401991 PMC3020049

[11] Van den Bruel A, Thompson M, Buntinx F, Mant D. Clinicians’ gut feeling about serious infections in children: observational study. British Medical Journal. 2012;345(7876):14–. 10.1136/bmj.e6144 PMC3458229 23015034

[12] Health Protection Agency. Health Protection in the 21st Century. Understanding the Burden of Disease; preparing for the future. London; 2005.

[13] Jones CHD, Neill S, Lakhanpaul M, Roland D, Singlehurst-Mooney H, Thompson M. The safety netting behaviour of first contact clinicians: a qualitative study. BMC Family Practice. 2013;14(1). 10.1186/1471-2296-14-140 PMC3849506 24066842

[14] Neill SJ, Coyne I. The Role of Felt or Enacted Criticism in Parents’ Decision Making in Differing Contexts and Communities: Toward a Formal Grounded Theory. Journal of Family Nursing. 2018;24(3):443–69. 10.1177/1074840718783488 29947565 PMC6094502

[15] Neill SJ. Containing acute childhood illness within family life: a substantive grounded theory. Journal of Child Health Care. 2010;14(4):327–44. 10.1177/1367493510380078 20823078

[16] Houston AM, Pickering AJ. 'Do I don't I call the doctor': a qualitative study of parental perceptions of calling the GP out-of-hours. Health Expectations. 2000;3(4):9. 10.1046/j.1369-6513.2000.00109.x PMC5060119 11281934

[17] NHS Institute for Innovation and Improvement Focus on Children and Young People Emergency and Urgent Care Pathway. Delivering on Quality and Value. Coventry; 2010.

[18] Moher D, Liberati A, Tetzlaff J, Altman DG, Group P, The PG. Preferred reporting items for systematic reviews and meta-analyses: the PRISMA statement. PLoS Medicine. 2009;6(7):e1000097 10.1371/journal.pmed.1000097 19621072 PMC2707599

[19] Emery DP, Milne T, Gilchrist CA, Gibbons MJ, Robinson E, Coster GD, et al The impact of primary care on emergency department presentation and hospital admission with pneumonia: a case-control study of preschool-aged children. NPJ Primary Care Respiratory Medicine. 2015;25(1):14113. 10.1038/npjpcrm.2014.113 PMC4498163 25654661

[20] Kilpi T, Anttila M, Kallio MJT, Peltola H. Severity of childhood bacterial meningitis and duration of illness before diagnosis. The Lancet. 1991;338(8764):406–9. 10.1016/0140-6736(91)91032-p 1678083

[21] McIntyre PB, MacIntyre CR, Gilmour R, Wang H. A population based study of the impact of corticosteroid therapy and delayed diagnosis on the outcome of childhood pneumococcal meningitis. Archives of Disease in Childhood. 2005;90(4):391–6. 10.1136/adc.2003.037523 15781931 PMC1720332

[22] Nadel S, Britto J, Booy R, Maconochie I, Habibi P, Levin M. Avoidable deficiencies in the delivery of health care to children with meningococcal disease. Journal of Accident & Emergency Medicine. 1998;15(5):298–303. 10.1136/emj.15.5.298 PMC1343165 9785154

[23] Okike IO, Ladhani SN, Anthony M, Ninis N, Heath PT. Assessment of healthcare delivery in the early management of bacterial meningitis in UK young infants: an observational study. BMJ Open. 2017;7(8):e015700 10.1136/bmjopen-2016-015700 28827241 PMC5724087

[24] Thompson MJ, Ninis N, Perera R, Mayon-White R, Phillips C, Bailey L, et al Clinical recognition of meningococcal disease in children and adolescents. The Lancet. 2006;367(9508):397–403. 10.1016/S0140-6736(06)67932-4 16458763

[25] Urbane UN, Gaidule-Logina D, Gardovska D, Pavare J. Value of parental concern and clinician's gut feeling in recognition of serious bacterial infections: a prospective observational study. BMC Pediatrics. 2019;19(1):219–. 10.1186/s12887-019-1591-7 31269915 PMC6607523

[26] Young N. The pre-hospital experiences of Samoan families who have had a child admitted to hospital with pneumonia: a qualitative investigation. Pacific Health Dialog. 2001;8(1):20–8. 12017824

[27] Hong QN, Pluye P, Fàbregues S, Bartlett G, Boardman F, Cargo M, et al Mixed Methods Appraisal Tool (MMAT) Version 2018: User Guide. Registration of Copyright (#1148552), Canadian Intellectual Property Office, Industry Canada [Internet]. 2018.

[28] Hong QN, Pluye P, Fàbregues S, Bartlett G, Boardman F, Cargo M, et al Improving the content validity of the mixed methods appraisal tool: a modified e-Delphi study. Journal of Clinical Epidemiology. 2019;111:49–59.e1. 10.1016/j.jclinepi.2019.03.008 30905698

[29] Hodkinson P, Argent A, Wallis L, Reid S, Perera R, Harrison S, et al Pathways to Care for Critically Ill or Injured Children: A Cohort Study from First Presentation to Healthcare Services through to Admission to Intensive Care or Death. PLoS ONE. 2016;11(1):e0145473 10.1371/journal.pone.0145473 26731245 PMC4712128

[30] Roland D, Jones S, Coats T, Davies F. Are Increasing Volumes of Children and Young People Presenting to Emergency Departments Due to Increasing Severity of Illness? Academic Emergency Medicine. 2017;24(4):503–4.

[31] Excellence NIfHaC. Bacterial meningitis and meningococcal septicaemia. Management of bacterial meningitis and meningococcal septicaemia in children and young people younger than 16 years in primary and secondary care NICE clinical guideline 102. Issued: June 2010 last updates: February 2015. London: National Institute for Health and Clinical Excellence; 2010. 31851447

[32] Excellence NIfHaC. Sepsis: recognition, diagnosis and early management. NICE guideline. Published: 13 July 2016 nice.org.uk/guidance/ng51. London: National Institute for Health and Clinical Excellence.; 2016 13th July 2016. Contract No.: NG51.

[33] Jones CHD, Neill S, Lakhanpaul M, Roland D, Singlehurst-Mooney H, Thompson M. Information needs of parents for acute childhood illness: determining ‘what, how, where and when’ of safety netting using a qualitative exploration with parents and clinicians. BMJ Open. 2014;4(1):e003874 10.1136/bmjopen-2013-003874 24430877 PMC3902331

[34] Maguire S, Ranmal R, Komulainen S, Pearse S, Maconochie I, Lakhanpaul M, et al Which urgent care services do febrile children use and why? Archives of Disease in Childhood. 2011;96(9):810–6. 10.1136/adc.2010.210096 21642270

[35] Roland D, Jones C, Neill S, Thompson M, Lakhanpaul M. Safety netting in healthcare settings: what it means, and for whom? Archives of Disease in Childhood (Education & Practice Edition). 2014;99(2):48–53. 24164728 10.1136/archdischild-2012-303056

[36] Neill SJ, Jones CHD, Lakhanpaul M, Roland DT, Thompson MJ, team ASr, et al Parent's information seeking in acute childhood illness: what helps and what hinders decision making? Health Expectations. 2015;18(6):3044–56. 10.1111/hex.12289 25327454 PMC5810715

[37] Callery P. Maternal knowledge and professional knowledge: co-operation and conflict in the care of sick children. International Journal of Nursing Studies. 1997;34(1):27–34. 10.1016/s0020-7489(96)00033-8 9055118

[38] Costet Wong A, Claudet I, Sorum P, Mullet E. Why Do Parents Bring Their Children to the Emergency Department? A Systematic Inventory of Motives. International Journal of Family Medicine. 2015;2015:978412–10. 10.1155/2015/978412 26618002 PMC4649091

[39] Hummel K, Mohler MJ, Clemens CJ, Duncan B. Why Parents Use the Emergency Department During Evening Hours for Nonemergent Pediatric Care. Clinical Pediatrics. 2014;53(11):1055–61. 10.1177/0009922814540988 24990368

[40] Benchimol EI, Fortinsky KJ, Gozdyra P, Van den Heuvel M, Van Limbergen J, Griffiths AM. Epidemiology of pediatric inflammatory bowel disease: a systematic review of international trends. Inflammatory Bowel Diseases. 2011;17(1):423–39. 10.1002/ibd.21349 20564651

[41] Morrison AKMD Myrvik MPP, Brousseau DCMDMS Hoffmann RGP, Stanley RMMDM. The Relationship Between Parent Health Literacy and Pediatric Emergency Department Utilization: A Systematic Review. Academic Pediatrics. 2013;13(5):421–9. 10.1016/j.acap.2013.03.001 23680294 PMC3808118

[42] Ehrich K. Reconceptualizing ‘Inappropriateness’: Researching Multiple Moral Positions in Demand for Primary Healthcare. Health. 2003;7(1):109–26.

[43] Lass M, Tatari CR, Merrild CH, Huibers L, Maindal HT. Contact to the out-of-hours service among Danish parents of small children—a qualitative interview study. Scandinavian Journal of Primary Health Care. 2018;36(2):216–23. 10.1080/02813432.2018.1459431 29633663 PMC6066288

[44] Jones CHD, Ward A, Hodkinson PW, Reid SJ, Wallis LA, Harrison S, et al Caregivers' Experiences of Pathways to Care for Seriously Ill Children in Cape Town, South Africa: A Qualitative Investigation. PLoS ONE. 2016;11(3):e0151606 10.1371/journal.pone.0151606 27027499 PMC4814040

[45] Ertmann RK, Reventlow S, Söderström M. Is my child sick? Parents' management of signs of illness and experiences of the medical encounter: Parents of recurrently sick children urge for more cooperation. Scandinavian Journal of Primary Health Care. 2011;29(1):23–7. 10.3109/02813432.2010.531990 21080763 PMC3347933

[46] Carter B, Arnott J, Simons J, Bray L. Developing a Sense of Knowing and Acquiring the Skills to Manage Pain in Children with Profound Cognitive Impairments: Mothers' Perspectives. Pain Research and Management. 2017;2017:2514920 10.1155/2017/2514920 28458591 PMC5385219

[47] Ninis N, Phillips C, Bailey L, Pollock JI, Nadel S, Britto J, et al The role of healthcare delivery in the outcome of meningococcal disease in children: case-control study of fatal and non-fatal cases. BMJ. 2005;330(7506):1475 10.1136/bmj.330.7506.1475 15976421 PMC558454

[48] Pollard AJ, Nadel S, Ninis N, Faust SN, Levin M. Emergency management of meningococcal disease: eight years on. Archives of Disease in Childhood. 2007;92(4):283–6. 10.1136/adc.2006.102384 17376933 PMC2083684

[49] Haj-Hassan TA, Thompson MJ, Mayon-White RT, Ninis N, Harnden A, Smith LFP, et al Which early 'red flag' symptoms identify children with meningococcal disease in primary care? The British Journal of General Practice. 2011;61(584):e97–e104. 10.3399/bjgp11X561131 21375891 PMC3047346

[50] Verbakel J, Bruel A, Thompson M, Stevens R, Aertgeerts B, Oostenbrink R, et al How well do clinical prediction rules perform in identifying serious infections in acutely ill children across an international network of ambulatory care datasets? BMC Medicine. 2013;11(1):10–. 23320738 10.1186/1741-7015-11-10 PMC3566974

[51] Verbakel JY, Lemiengre MB, De Burghgraeve T, De Sutter A, Aertgeerts B, Shinkins B, et al Should all acutely ill children in primary care be tested with point-of-care CRP: a cluster randomised trial. BMC Medicine. 2016;14(1):131 10.1186/s12916-016-0679-2 27716201 PMC5052874

[52] Brent AJ, Lakhanpaul M, Thompson M, Collier J, Ray S, Ninis N, et al Risk score to stratify children with suspected serious bacterial infection: observational cohort study. Archives of Disease in Childhood. 2011;96(4):361–7. 10.1136/adc.2010.183111 21266341 PMC3158666

[53] Galetto-Lacour A, Zamora SA, Andreola B, Bressan S, Lacroix L, Da Dalt L, et al Validation of a laboratory risk index score for the identification of severe bacterial infection in children with fever without source. Archives of Disease in Childhood. 2010;95(12):968–73. 10.1136/adc.2009.176800 20515973

