# Supplementary material for: A systematic review of the organizational, environmental, professional and child and family factors influencing the timing of admission to hospital for children with serious infectious illness
Source: PLoS One. 2020 Jul 23;15(7):e0236013. doi: 10.1371/journal.pone.0236013 (PMC7377491; doi:10.1371/journal.pone.0236013)
Supplement: S1 File — (DOCX) [file pone.0236013.s001.docx]

**Supporting Information File 1: Definitions of serious infectious illness, developed from (11, 52, 53).**

| **Diagnosis** | **Criteria** |
| --- | --- |
| Pneumonia | Respiratory symptoms and signs and focal consolidation on X-ray reported by a paediatric radiologist. |
| Bronchiolitis | Severe bronchiolitis requiring PHDU care or PICU transfer. |
| Bacteraemia | Identification of a significant bacterial pathogen in blood using culture or molecular methods |
| Urinary tract infection | Growth of a single bacterial urinary tract pathogen at ≥10^5^ colony-forming units/ml in an appropriate urine sample in the context of clinical signs of systemic involvement. |
| Meningitis | Identification of a pathogen in CSF using culture or molecular methods, or clinical meningitis plus a cerebrospinal fluid analysis suggestive of viral or bacterial meningitis |
| Osteomyelitis | Clinical signs, and radiological confirmation of bone changes |
| Septic arthritis | Isolation of a bacterial pathogen from a joint using culture or molecular methods |
| Probable SBI | Clinical signs of infection with at least 2 NICE sepsis high risk criteria, resuscitation>20mls/kg; or PHDU/PICU care  Clinical signs of infection on admission and prolonged admission, and administration of intravenous antibiotics beyond 72h despite negative culture results. |
| Encephalitis | Clinical signs and contributory evidence on CSF analysis/ neuroimaging |
| Cellulitis | Acute, diffuse, spreading infection of the skin, involving the deeper layers of the skin and the subcutaneous tissue. |
| Suppurative cervical lymphadenitis | Enlarged, inflamed and tender lymph node with or without fluctuance, usually unilateral. Clinical diagnosis is confirmed by positive culture or Gram stain of specimen obtained by needle aspiration or incision and drainage. |
| Pre-septal & post-septal cellulitis | Pre-septal cellulitis (periorbital cellulitis) is infection of the eyelid and surrounding skin anterior to the orbital septum. Orbital cellulitis (post-septal cellulitis) is infection of the orbital tissues posterior to the orbital septum. |
| Eczema herpeticum^[[1]](#footnote-1)^ | Clinical diagnosis and/or positive culture or molecular methods |
| Deep head and neck infections | Suppurative infection of the neck, including:  Peritonsillar abscess: Collection of pus located between the capsule of the palatine tonsil and the pharyngeal muscles.  Retropharyngeal abscess: Collection of pus located in the retropharyngeal space (extending from the base of the skull to the posterior mediastinum, between the middle layer and the deep layer of the deep cervical fascia).  Lateral pharyngeal space infection: Collection of pus located in the lateral pharyngeal space (bounded laterally by the carotid sheath). |
| Endocarditis | Clinical concerns and Duke’s criteria |

1. We included Eczema herpeticum as it is considered a medical emergency, and a high index of suspicion is required. Prompt treatment with oral acyclovir should be initiate, and in immunocompromised patients, hospitalization for intravenous antivirals is required. Eczema herpeticum has a reported mortality of up to 10% if untreated. It mostly presents in a localised form, but rarely can disseminates via haematogenous spread with pulmonary, hepatic, ocular and neurological manifestations. 54. Finlow C, Thomas J. Disseminated herpes simplex virus: a case of eczema herpeticum causing viral encephalitis. The Journal of the Royal College of Physicians of Edinburgh. 2018;48(1):36-9. [↑](#footnote-ref-1)
