# Supplementary material for: A systematic review of the organizational, environmental, professional and child and family factors influencing the timing of admission to hospital for children with serious infectious illness
Source: PLoS One. 2020 Jul 23;15(7):e0236013. doi: 10.1371/journal.pone.0236013 (PMC7377491; doi:10.1371/journal.pone.0236013)
Supplement: S2 File — (DOCX) [file pone.0236013.s002.docx]

**Supporting Information File 2: Search conducted in MEDLINE via EBSCOHost**

|  |
| --- |

| **#** | **Query** | **Limiters/Expanders** | **Last Run Via** | **Results** |
| --- | --- | --- | --- | --- |
| S23 | S1 AND S2 AND S3 AND S4 AND S15 AND S22 | Search modes - Boolean/Phrase | Interface - EBSCOhost Research Databases  Search Screen - Advanced Search  Database - MEDLINE | **413** |
| S22 | S16 OR S17 OR S18 OR S19 OR S20 OR S21 | Search modes - Boolean/Phrase | Interface - EBSCOhost Research Databases  Search Screen - Advanced Search  Database - MEDLINE | **964,545** |
| S21 | First contact* | Search modes - Boolean/Phrase | Interface - EBSCOhost Research Databases  Search Screen - Advanced Search  Database - MEDLINE | **6,777** |
| S20 | Community OR Primary care | Search modes - Boolean/Phrase | Interface - EBSCOhost Research Databases  Search Screen - Advanced Search  Database - MEDLINE | **750,694** |
| S19 | NHS111 OR telephone service OR telephone triage OR helpline | Search modes - Boolean/Phrase | Interface - EBSCOhost Research Databases  Search Screen - Advanced Search  Database - MEDLINE | **6,268** |
| S18 | Ambulance service | Search modes - Boolean/Phrase | Interface - EBSCOhost Research Databases  Search Screen - Advanced Search  Database - MEDLINE | **2,524** |
| S17 | Ambulatory care OR Urgent care OR Emergency care | Search modes - Boolean/Phrase | Interface - EBSCOhost Research Databases  Search Screen - Advanced Search  Database - MEDLINE | **88,237** |
| S16 | Out of hours OR After hours | Search modes - Boolean/Phrase | Interface - EBSCOhost Research Databases  Search Screen - Advanced Search  Database - MEDLINE | **136,422** |
| S15 | S5 OR S6 OR S7 OR S8 OR S9 OR S10 OR S11 OR S12 OR S13 OR S14 | Search modes - Boolean/Phrase | Interface - EBSCOhost Research Databases  Search Screen - Advanced Search  Database - MEDLINE | **4,585,551** |
| S14 | Timely treatment OR Rapid management | Search modes - Boolean/Phrase | Interface - EBSCOhost Research Databases  Search Screen - Advanced Search  Database - MEDLINE | **7,317** |
| S13 | Health service OR health systems | Search modes - Boolean/Phrase | Interface - EBSCOhost Research Databases  Search Screen - Advanced Search  Database - MEDLINE | **592,423** |
| S12 | Safety netting OR Information seeking OR information giv*OR Recognising symptoms OR health seeking | Search modes - Boolean/Phrase | Interface - EBSCOhost Research Databases  Search Screen - Advanced Search  Database - MEDLINE | **12,497** |
| S11 | Preventable OR increased OR decreased AND morbidity OR mortality | Search modes - Boolean/Phrase | Interface - EBSCOhost Research Databases  Search Screen - Advanced Search  Database - MEDLINE | **3,514,752** |
| S10 | Barriers to healthcare OR access to health* | Search modes - Boolean/Phrase | Interface - EBSCOhost Research Databases  Search Screen - Advanced Search  Database - MEDLINE | **27,921** |
| S9 | Earl* diagnosis OR Late diagnosis OR Missed opportunities OR Recognition OR earl* intervention OR interpretation of symptoms OR identification of symptoms | Search modes - Boolean/Phrase | Interface - EBSCOhost Research Databases  Search Screen - Advanced Search  Database - MEDLINE | **462,912** |
| S8 | Tim* of referral OR del* referral OR late referral | Search modes - Boolean/Phrase | Interface - EBSCOhost Research Databases  Search Screen - Advanced Search  Database - MEDLINE | **8,387** |
| S7 | Tim* of assessment OR dela* assessment OR late assessment | Search modes - Boolean/Phrase | Interface - EBSCOhost Research Databases  Search Screen - Advanced Search  Database - MEDLINE | **34,192** |
| S6 | Tim* of treatment OR dela* treatment OR late treatment OR earl* treatment OR timely treatment OR timely consultation OR dela* consultation | Search modes - Boolean/Phrase | Interface - EBSCOhost Research Databases  Search Screen - Advanced Search  Database - MEDLINE | **268,738** |
| S5 | Tim* of admission OR dela* admission OR late presentation OR deter presentation OR dela* presentation | Search modes - Boolean/Phrase | Interface - EBSCOhost Research Databases  Search Screen - Advanced Search  Database - MEDLINE | **24,975** |
| S4 | General practi* OR Health visitor OR Paramedic* OR Family doctor OR Family physician OR Nurse OR Practice nurse OR Community children’s nurse OR P?diatric nurse OR children’s Nurse | Search modes - Boolean/Phrase | Interface - EBSCOhost Research Databases  Search Screen - Advanced Search  Database - MEDLINE | **390,500** |
| S3 | Serious infection* OR Septi* or Sepsis OR Pneumonia OR mening* OR encephalitis OR Respiratory OR Severity of illness | Search modes - Boolean/Phrase | Interface - EBSCOhost Research Databases  Search Screen - Advanced Search  Database - MEDLINE | **975,981** |
| S2 | Child* OR infant* or bab* or P?diatric* | Search modes - Boolean/Phrase | Interface - EBSCOhost Research Databases  Search Screen - Advanced Search  Database - MEDLINE | **2,549,014** |
| S1 | Family OR families OR parent* OR caregiver* OR caretaker OR carer* OR mother OR father | Search modes - Boolean/Phrase | Interface - EBSCOhost Research Databases  Search Screen - Advanced Search  Database - MEDLINE | **1,513,905** |
