# Supplementary material for: A systematic review of the organizational, environmental, professional and child and family factors influencing the timing of admission to hospital for children with serious infectious illness
Source: PLoS One. 2020 Jul 23;15(7):e0236013. doi: 10.1371/journal.pone.0236013 (PMC7377491; doi:10.1371/journal.pone.0236013)
Supplement: S3 File — (DOCX) [file pone.0236013.s003.docx]

**Supporting Information File 3: Detailed Data Extraction Summary**

| **Author, year, Country** | **Aim** | **Study Design, Methods** | **Recruitment, Participant Numbers: Participated** | **Child: Age, Gender, Socioeconomic Status (SES), Disease Characteristics, Other.  Parents: Age, Gender** | **Help-seeking behaviours, Organisational factors, Environmental factors, SES, Other findings.** | **Types of bias reported, Limitations stated, Populations excluded** |
| --- | --- | --- | --- | --- | --- | --- |
| Crocker (2013) UK [7] | **I**dentify differences between children who consulted a General Practitioner (GP) and children who did not consult a GP before the day of hospital presentation with pneumonia or empyema | **Design:** Mixed methods sequential sub sample design. **Methods:** self-completed questionnaire by parent/carers; interviews with sub-set of parents; patient hospital record review. | **Recruitment:** Purposive. Recruiting clinicians approached carers of all eligible patients. In-patients from 7 hospitals with inpatient paediatric units in South Wales. **Participated:** N=151 (n=94 early consulters; n=57 late/non-consulters) completed questionnaire; subset (n=28) were interviewed. | **Age**: 6 months-16 years (mean 5 years). **Gender**: 59.6% male for early consulters, 56.1% male for late and non-consulters.  **SES:** All quintiles represented (measured using Welsh Multiple Index of Deprivation).  **Disease**: Community acquired pneumonia (86.1%) or empyema (13.9%).  **Other:** Among early consulters: mean age 3.82 years, 59.6% were male, 92.6% of white ethnicity. Among late and non-consulters: mean age 4.19 years and 56.1% were male and 96.5% were of white ethnicity  **PARENT: Gender:** Carer gender not recorded | **Help-seeking behaviours:** Late/non-consulters significantly less likely to have taken antibiotics before presenting to hospital, & significantly more likely to have obtained advice from the NHS Direct Telephone helpline and website and had significantly more rapid onset of illness.  Among interviewed parents who had not seen General Practitioner (GP) prior factors included: did not consider consulting a GP earlier; did not think earlier symptoms were serious/unusual due to child initially improving; presumed asthma; thought abdominal pain was pulled muscle; child was alert/active; child's breathing was normal; & presumed common self-limiting infection (3.6%).  **Organisational**: Factors for no GP presentation included: GP surgery was closed, difficulty getting a GP appointment, negative perceptions (including afraid of wasting doctor's time and anticipating GP would diagnose a viral infection).  **Environmental**: Factors for late/no GP presentation: unable to travel to GP surgery and GP declined home visit (child too ill to move and carer had no car). **SES:** Late/non-consultation associated with lack of home ownership, WIMD quintile and higher ratio of children to adults in the household. | **Bias:** Invitation bias, response bias, selection bias, recall bias. **Excluded populations:** Children who saw a General Practitioner for pneumonia but never presented to hospital were not included so this excluded those likely to have had with less severe pneumonia. |
| Emery (2015), New Zealand [19] | **I**dentify primary care factors associated with Emergency Department presentation and hospital admission of preschool-aged children with community-acquired pneumonia. | **Design:** Mixed methods (part of larger case=control study). **Methods:** Modified General Practice Assessment Survey; primary care review; interview; & hospital record review. | **Recruitment:** All eligible children approached in hospital setting. Controls randomly selected from the community. **Participated**: N=856: hospitalised with pneumonia (n=326); pneumonia discharged from ED (n=179); controls (n=351). | **Age**: <5 years old (mean 19 months).  **SES:** Measured by household deprivation score.  **Disease:** Pneumonia (acute illness with cough or breathing difficulty plus in-drawing or tachypnoea).  **Other:** Children admitted with pneumonia younger than those with pneumonia discharged from ED. Children with pneumonia had lower weight for height z-scores than community controls | **Help-seeking behaviours:** Children less likely to present to ED with pneumonia included: those whose caregivers would take them back to the same doctor, or to an after-hours accident and medical centre when still unwell with a cough and fever, following visit to General Practitioner (GP) on previous day. Increased likelihood of ED presentation with pneumonia was associated with lower parental satisfaction scores for continuity of care, communication between the caregiver and GP & overall caregiver satisfaction with care. Children who had made more health professional visits before presentation had an increased likelihood of admission with pneumonia. Children whose caregiver would seek care from a complementary healer if child was unwell with a cough or fever less likely to be admitted with pneumonia. Children whose caregivers would take them to a hospital ED if they had been seen the previous day by their GP and were still unwell less likely to be admitted with pneumonia.  **Organisational:** Children more likely to present with pneumonia included: those without a single, identified GP, whose GP worked part-time ⩽20hr week, whose GP had no immunisation recall. Likelihood of hospital admission with pneumonia increased when child's GP belonged to a Primary Health Organisation, when antibiotics were prescribed by the GP before ED presentation. Referral by a health professional compared with self-referral was associated with an increased likelihood of admission with pneumonia. Children without a single, identified GP were less likely to be admitted with pneumonia. | **Bias:** Seasonal bias, uneven distribution. |
| Francis (2011), UK [6] | Identify potential opportunities for earlier intervention among young children who develop a complicated respiratory tract infection (RTI). | **Design:** Qualitative interview study. **Methods:** Interviews (guided)**.** | **Recruitment:** Purposive. Parents of all eligible children approached at a large teaching hospital (University Hospital of Wales). **Participated**: N=22. | **Age:** 16 months-13yrs (median 4yrs).  **Gender**: female (n=12), male (n=10):  **SES:** Not reported but no wage earners (n=6), at least one PT wage earner (n=16).  **Disease**: Empyema (n=12), pneumonia (n=8), peritonsillar abscess (n=1), mastoiditis and lateral sinus thrombosis (n=1).  **PARENT:** **Gender:** Mothers (n=22), father (n=1) | **Help-seeking behaviours:** Difficulties in assessing the severity of their child’s illness or interpreting their child’s symptoms. All parents described potentially serious symptoms (most common: rapid breathing, panting or grunting). Although most regarded these symptoms as unusual/worrying, nearly half described delay of 24h or more between first identifying the symptom(s) and consulting a healthcare professional (HCP). Parents not consulting at an earlier time because of a fear of ‘overreacting’ and not wanting to ‘bother’ service or based on past experience, they did not believe their concerns would be taken seriously. One parent delayed consulting because believed antibiotics would be prescribed too readily,  one did not take child to an out-of-hours service as they would not know child’s past history, and one whose child was experiencing difficulty breathing, did not feel she was allowed to take her child directly to Emergency Department.  **Organisational (parent reported)**: Delays included: difficulties with General Practitioner (GP) appointment system, e.g., difficulties in getting through on the telephone, prolonged waits for emergency appointments, being told that there were no more emergency appointments that day, and having to wait for a doctor to call them back, sometimes hours later in the day. Failures/problems of appropriate triage included; inadequate telephone triage by practice nurse, parent felt incapable of assessing features of her child’s illness. Failures of HCPs to respond appropriately after child had developed one or more serious symptoms (rapid breathing, panting, grunting); prescribed antibiotics over the phone (without being seen), even though the child had developed breathlessness and panting since a previous consultation the week before. Parent not using a delayed prescription because confused by the doctor’s advice that this was ‘a viral infection’ and antibiotics were ‘a precaution’. Feeling dismissed after consulting in primary care, out-of-hours, Emergency Department and secondary care settings. Other sources of being dismissed: advised to give child over-the-counter medicines, belief child had been inadequately examined, leaving the consultation with a poor understanding of their child’s illness. Frustration at the outcome of their consultations: consultations rushed, unable to challenge clinical decisions. | **Bias:** Retrospective bias. **Limitations**: Parents who had problems in pre-hospital management of child's illness might have been more likely to agree to participate. Data on those not recruited not available.  **Excluded populations:** Only one child with peritonsillar abscess, one mastoiditis and no cases of acute rheumatic fever included, so findings may not be relevant to children with these complications. |
| Grant (2012), New Zealand [9] | Describe primary care antibiotic use for children subsequently hospitalised with community acquired  pneumonia. | **Design:** Case series. **Methods:** Case series review and parents asked to describe child’s symptoms. Hospital medical records reviewed. | **Recruitment:** Children with pneumonia were identified from daily review of admissions at children’s hospital in NZ **Participated**: N=280. | **Age:** <5 years (median 17 months, IQR 9-26 months).  **Gender:** 45% female. **SES:** Households in socioeconomically deprived areas over-represented (34% in bottom quintile) using NZ Index of Social Deprivation.  **Disease**: pneumonia.  **Other:** Of 220 who had opportunity to receive antibiotics, severe pneumonia (n=202), mild pneumonia (n=8). | **Other findings:** Receipt of antibiotic more likely if child seen by own General Practitioner (GP), less likely if the primary care clinician failed to make a diagnosis of LRTI. Mild pneumonia associated with increased likelihood of being prescribed antibiotics.  Children with no opportunity to receive antibiotics had more rapidly evolving illness than those with opportunity to receive antibiotics. Reasons for missed opportunity to receive antibiotic: 102/112 made at least one GP visit, 23/112 prescription written but not collected, 2 diagnosed pneumonia/bronchopneumonia but no antibiotic prescribed. Of remaining children, 28 presented with increased respiratory rate or breathing difficulty, other respiratory symptoms (cough, wheeze, noisy breathing) n=23, systemic symptoms (vomiting, poor feeding, increased sleeping, irritability, lethargy) n=49. 41 had respiratory and systemic presenting symptoms of which 30% did not have missed LRTI diagnosis in primary care | **Bias:** recall bias. **Excluded populations:** Only included children admitted to hospital therefore not possible to determine if primary care antibiotic treatment reduces risk of hospital admission |
| Kilpi (1991), Finland [20] | See whether duration of illness in childhood cases of bacterial meningitis before admission to hospital was related to the severity of illness | **Design:** Prospective cases series. **Methods:** Questionnaire (parent); case note review. | **Recruitment:** Purposive. n-patients,18 paediatric hospitals in Finland. **Participated:** N=286 eligible for analysis. | **Age:** 3 months-15 years (mean 2.9yrs, SD 3.2).  **Gender:** male, 54%. **SES:** Not reported.  **Disease:** Bacterial meningitis; haemophilius influenzae type b (69%); Nesseria meningitis (23%) streptococcus penumoniae (6%); Group B streptococcus (1%) Listeria monocytogenes (0.5%); E coli (0.5%).  **Other**: Children with short duration of illness (<24h, n=141); intermediate duration (>24-48h, n=75); long duration (>48h, n=70). | **Other findings:** Level of consciousness significantly poorer in children with short history of illness than those with long history. Seizures before or on admission were more common in the short history (24%) than the intermediate (19%) or long history (9%) groups. Children with long history of illness significantly younger than those ill for up to 48hr. Full consciousness more common in children of 3 years or older than in the younger ones. Unconsciousness was equally common in the three age groups and in each group the children with the longest duration of symptoms did best. Infants had seizures slightly more often (26%) than older children (17%). Seizures not associated with duration of illness in the youngest age group, whereas in the two older age groups seizures occurred exclusively among children with a history of 48hr or less. | Not stated |
| McIntyre (2005), Australia [21] | Determine independent contribution of corticosteroid therapy and delayed diagnosis on outcome of childhood pneumococcal meningitis. | **Design:** Case series (lab and hospital). **Methods:** Case note review; questionnaire **(**paediatrician/family physician). | **Recruitment:** Population register established through laboratories in the study area. Laboratories and hospital surveillance system in Sydney Australia. **Participated**: N=122 eligible cases. | **Age:** 1.78-179 months (median 13 months).  **Gender:** male, 62%. **SES:** Not reported.  **Other**: Most cases (46.3%) occurred in children <5yrs. 1st presentation: hospital (40%), General Practitioner (GP) (60%). Of GP group, 32% had two or more presentations before admission to hospital. 12% whose first presentation was to hospital had more than one attendance. In 46% cases, hospital admission occurred more than 24 hours after the time of onset of symptoms recorded in the clinical records. | **Other findings:** Significant diagnostic and prognostic predictors of outcome were not having a lumbar puncture done, intensive care admission, intubation, any neurological abnormality, seizures within 48 hours, and higher temperature. The only significant therapeutic factor was administration of corticosteroids with or before antibiotics. | **Limitations**: Some clinical data only available from medical records due to incomplete physician follow up, might mean underestimation of mild disability due to lesser degrees of morbidity not being detected at hospital or uncovered later. |
| Nadel (1998), UK [22] | Evaluate to frequency of delays and suboptimal management in children with meningococcal disease | **Design:** Prospective case note review. **Methods: D**irect questioning (parents) & General Practitioner (GP) referral letters. Case note review. | **Recruitment:** Purposive. First 54 in-patients diagnosed at tertiary paediatric department within the study period. **Participated**: N=54 | **Age:** 1 week-15.7yrs (median 2.95 yrs).  **Gender**: male (n=31), female (n=23).  **SES:** Not reported.  **Disease:** Meningococcal septicaemia (n=42), meningococcal meningitis (n=12). Of those with septicaemia, 15 also had clinical or lab evidence of meningitis. | **Help-seeking behaviours:** Some parents delayed presentation due to hesitation to call General Practitioner (GP) or go to Emergency Department at night/ weekend, not appreciating significance of non-blanching rash, inappropriately reassured by advice over phone by Emergency Department or GP. In all cases, parents were unaware of signs of serious illness in their child.  **Other findings:** Among children with septicaemia delay from onset until treatment initiation was longer for those who died compared with survivors. 65% children had delayed recognition or suboptimal treatment: typical clinical features were referred to hospital without receiving parenteral penicillin from GP (n=8), not recognised despite presence of fever, petechial/purpuric rash and clinical features of serious illness and not treated or referred (n=16). Delay between presentation and initiation of treatment was delayed 2-12 hours (median 8.5). Repeated visits to GP (n=9), taken to Emergency Department without further attempts to see GP (n=7).  Children presented directly to Emergency Department (n=17): typical clinical features on presentation (n=15). In 2, incorrect diagnosis made and antibiotic treatment withheld and in 1 treatment was delayed by 4 hours despite diagnosis – across 3 treatment delay was 2.5-12 hours. 71% had appropriate treatment in Emergency Department.  All 54 admitted into hospital. | **Bias:** recall bias. **Limitations**: Debate about reliability of the signs of serious illness in children used in this study. |
| Okike (2017), UK [23] | Define early presenting features of bacterial meningitis in young infants in England and review the adequacy of individual case management compared with relevant national guidelines and expert panel review. | **Design:** Retrospective medical case note review **Methods:** Questionnaire (parent); case note review. | **Recruitment:** Purposive. 48 hospitals representing all English regions. **Participated**: N=97 cases included in analysis. | **Age:** <90 days (0-6 days 31%, 7-28 days 45%, 29-89 days 24%).  **Gender:** male 54%.  **SES:** Parental accommodation: Mothers 52% own home, 40% rented, 8% council home, Fathers 58% own home, 34% rented, 8% council.  **Disease**: Group B strep (63%), E Coli (11%), Listeria monocytogenes (4%), Neisseria meningitis (4%), Other gram-negative bacteria (10%), Other gram-positive bacteria (5%). **Other**: admitted from home (68%), inpatient (32%). Median age higher among cases admitted from home (17 days) compared with those already in hospital (1 day).  **PARENT: Age:** (median) mothers 29yrs; fathers 32yrs. | **Help-seeking behaviour:** 20 parents took infants straight to the hospital (Emergency Department or urgent care centre); remainder phoned General Practitioner (GP) or 24-hour NHS Direct telephone service or contacted community midwife; of these, 28% advised to stay at home. Most common features at onset of illness were poor feeding, lethargy and fever. Median time from onset of 1st features to 1st help was 5.0 hours. Majority of parents presented to hospital within 24 hours of onset of symptoms. 93% of infants who presented after 24 hours had fever or seizures or both at time they presented to hospital; of these 53% had attended their GP surgery before going to hospital, and, of these, three were reviewed at the Emergency Department/walk-in centre and sent home and two were initially seen by a community midwife. Remaining 47% infants were brought to hospital by their parents more than 24 hours from the onset of symptoms. 30% infants were assessed to have received inappropriate prehospital management. 12 infants with fever warranted further investigation according to the NICE guidelines and in 8, there was a delay in seeking help despite the presence of worrying clinical features.  **Organisational:** uncertainty in recognition, over-reliance on the presence of fever, waiting for urine samples before giving antibiotics and waiting for handover between shifts.  **Other findings:** 55% infants triaged in Emergency Department during normal working hours. 68% infants had onset of symptoms within 72 hours of birth and therefore assessed against the NICE early-onset antibiotic guidelines. The main differences between infants admitted from home and inpatient cases were age, presence of fever on presentation, timing of lumbar puncture and time to discharge from outpatient follow-up. | **Bias:** recall bias. **Limitations**: Parents recruited at paediatrician’s discretion. Some parents may have agreed to participate simply because they were concerned about their child's long term outlook or about suboptimal healthcare. |
| Thompson (2006), UK [23] | Systematic assessment of the sequence and development of early symptoms of meningococcal disease before admission to  hospital. | **Design:** Observational study. **Methods:** Questionnaire (parent); interviews (General Practitioner (GP)); case note review. | **Recruitment:** Purposive. UK hospitals & GP records. **Participated:** N=448 children with meningococcal disease, 103 died. | **Age:** ≤16yrs.  **SES:** Not reported  **Disease:** Meningococcal disease (of the 448 children with meningococcal disease, 103 died). | **Help-seeking behaviour:** 51% of children seen by General Practitioner (GP) were sent to hospital from the 1st consultation. In most children, the disease progressed very rapidly. The median time between onset and admission to hospital was 22h in the oldest children (15–16yrs) and less in younger children (13h in those younger than 1 year, 14h in those aged 1–4yrs, 20 h in those aged 5–14yrs). 25% children had symptoms in the two weeks before the onset of meningococcal disease, most of which were suggestive of URTI or LRTI. Only 7% children had seen a doctor in the week before the onset of disease. 76·1% parents had noticed 1/more of early symptoms before hospital admission; <10% children presented with classic signs of meningism or impaired consciousness without parents having previously recognised a haemorrhagic rash or early signs of sepsis.  **Other findings:** Fever was 1st symptom to be noticed in children <5yrs; headache 1st to be seen in those >5yrs. Loss of appetite, nausea, and vomiting were early features for all age groups, with many children also having upper respiratory symptoms (sore throat and coryza). In all age groups, 1st specific clinical features that were signs of sepsis: leg pain, abnormal skin colour, cold hands and feet, and, in older children, thirst. Parents of younger children also reported drowsiness and difficulty in breathing and occasionally diarrhoea. Most sepsis symptoms occurred before the first medical contact. 1st classic symptom of meningococcal disease to emerge was rash. Median time of onset of specific meningitis symptoms (neck stiffness, photophobia, bulging fontanelle) was later, around 12–15h from onset of illness. Last signs (such as unconsciousness, delirium, or seizures) were seen at a median of 15h in infants (under 1yr), and about 24h in older children. Features that were present in more than half the children before admission to hospital were nonspecific (i.e., common in self-limiting viral illness) except for haemorrhagic rash, which emerged late (median 13h). 3 features of sepsis (leg pain, abnormal skin colour and cold hands and feet) occurred earlier in the illness. Median time of onset of the classic meningococcal features of haemorrhagic rash, meningism, and impaired consciousness was 13–22h. By contrast, the median time of onset of the early, non-specific symptoms was 7–12h. | **Bias:** recall bias. **Limitations**: retrospective data, no data for children with other illnesses to compare symptom frequency. |
| Urbane (2019), Latvia [25] | Assess the diagnostic value of parental concern and gut feeling at the Emergency Department of a tertiary hospital | **Design:** Prospective observational study. **Methods:** Questionnaire (clinicians, parent); case note review. | **Recruitment:** Purposive. ED of children's clinical University hospital in Riga. **Participated**: N=162. | **Age**: 2 months -17.8 years (median 43.5 months); < 1yr (n=22); 1-5yrs (n=80).  **Gender**: male 53.1%(n=86). **SES**: Not reported.  **Disease**: serious bacterial infection present in 46 patients: UTI 4.3%, sepsis 1.9%, pneumonia 17.3%, acute osteomyelitis with bacteraemia 0.6%, bacterial meningitis with bacteraemia 0.6%, bacterial gastroenteritis 1.9%, acute appendicitis 1.2%, bacterial soft tissue infection 0.6%. Serious bacterial infection absent 71.6%.  **PARENT: Age:** mothers median 34yrs; fathers median 33yrs.  **Gender**: Mothers, 88.3%. | **Help-seeking behaviour:** 59.9% parents stated belief that fever itself is indicative of serious illness, some parents believed that other symptoms must be considered as well when evaluating the severity of illness, few parents did not believe that fever is indicative of serious illness. No association was found between the belief that fever is indicative of serious illness and parental concern.  **Other findings:** The presence of clinician’s “gut feeling” was significantly more common in children who developed serious bacterial infection than in those who did not, as was “sense of reassurance” in the cases with no serious bacterial infection. Prognostic value of “gut feeling” in ruling in/out possibility of being diagnosed with serious bacterial infection in study population not significant. Likelihood of the patient being diagnosed with serious bacterial infection was higher when “gut feeling” expressed by certified paediatricians than by paediatric residents. Sense of reassurance associated with decreased likelihood of having serious bacterial infection. The rule-out value of sense of reassurance was not significant. | **Limitations**: Non-representative of all febrile patients presenting to Emergency Department; sample size of parents providing consent small. Prevalence of serious bacterial infections non-representative of prevalence at study site. Parents of children in Emergency Department longer maybe more prone to consent. **Excluded populations:** Lower risk patients scarcely enrolled. |
| Van den Bruel (2012), Belgium [11] | Investigate the basis and added value of clinicians "gut feeling" that infections in children are more serious than suggested by clinical assessment | **Design:** Observational study. **Methods:** Case note review. | **Recruitment:** Prospective consecutive series, Primary care, Flanders, Belgium. **Participated**: N=3890. | **Age**: 0-16yrs (mean 5.05yrs). **Gender**: male, 51.4%. **SES:** Not reported. **Disease:** 21/3890 admitted to hospital with serious infection; pneumonia (n=12), pyelonephritis (n=6), sepsis, meningitis, cellulitis & bacterial lymphangitis (n=1).  **Other:** Of 3369 children assessed as non-serious illness, 6 subsequently admitted to hospital with serious infection. Of all children presenting to primary care, gut feeling present in 13/21 with serious & 107/3869 with non-serious infection. In children in whom clinical impression was non-serious illness (n=3369), gut feeling present in 2/6 with serious & 44/3363 with non-serious infection. | **Other findings:** Gut feeling that something was wrong despite clinical assessment of a non-serious illness increased risk of serious illness & acting on this feeling had potential to prevent to cases being missed at cost of 44 false alarms. Compared with clinical impression that the children were seriously ill, gut feeling was consistently more specific, irrespective of the children’s age or diagnosis or the seniority of the doctor.   Feature most strongly associated with gut feeling was a history of convulsions. Children’s appearance, pattern of breathing and level of drowsiness were also significant but much less likely to provoke gut feeling than parental concern. Temp did not influence gut feeling, history of cough or diarrhoea made it less likely. Weight loss and urinary symptoms were also independently associated with gut feeling. For every year of additional experience the clinician was 5% less likely to experience a gut feeling separate from their clinical assessment. Of the 21 children who were eventually admitted to hospital with a serious infection, nine were not referred at first contact (despite the initial clinical impression of a serious illness in four children) of these, mean age, mean duration of illness, & mean temperature did not differ significantly from those referred immediately at first contact. However, in four of the nine children (44%), the clinician had a gut feeling of something serious. | **Limitations**: Analysing components of gut feeling was not the primary aim of the original study and so study was not powered to do so. Possible bias related to the diagnostic investigations - children for whom clinicians had a gut feeling were referred more often that children for whom clinicians did not have a gut feeling. **Excluded populations:** only included children assessed in primary care and not other clinical settings. |
| Young (2001), New Zealand [26] | Describe the pre-hospital experiences; health actions and attitudes towards health services of Samoan families who have had  a child hospitalised with pneumonia. | **Design:** Qualitative. **Methods:** Interviews, open-ended, in-depth interviews. | **Recruitment:** Purposive. maximum variation selection. In-patients, Starship children's hospital. 11/12 families were from West Auckland. **Participated:** N=12. | **Age**: <2yrs.  **Gender**: not reported.  **SES**: Not reported.  **Disease:** Viral pneumonia (n=10), bacterial pneumonia (n=2).  **Other**: 6/8 referred were Samoan born and 2/4 self-referred were Samoan-born. | **Help-seeking behaviour:** Caregivers perceived themselves to be: quick to recognise change from normal in their child, prompt taking them to doctor, able to distinguish this illness from previous non-urgent illnesses, knew instinctively the child was unwell (“mothers' instinct”), felt these subjective feelings dismissed by the doctor leading to mistrust and influenced the caregiver to seek help elsewhere. All parents sent home after the initial consultation but quick to return to doctor if they felt their child was not improving. Personal barriers to accessing General Practitioner (GP) included: lack of knowledge about services in area, poor communication, feeling uncomfortable asking questions, contradictory information given by healthcare professionals (HCPs), feeling concerns dismissed as unimportant by HCPs in the community.  **Organisational**: Most caregivers visited 2 or more doctors in the community before being referred/self-referring, reasons included: mistrust of doctors, contradictory information given by different HCPs, & poor communication between HCPs & caregivers. Reasons for wanting to return to family doctor included being able to ask questions, feeling comfortable when communicating with the doctor and feeling concerns acknowledged as important.  **Environmental:** Non-financial barriers for attending accident/medical setting rather than GP included: GP fully booked, working mothers unable to attend within opening hours, limited transport to GP, the GP further than the accident/medical clinic, child's symptoms became concerning in evening and GP closed. | **Limitations:** some statements presented in paper appear to be assumptions made by author. |
